# Diagnostic yield of blood cultures in febrile neutropenia—a real-world observational study from an academic medical center during blood culture bottle shortage

**DOI:** 10.1017/ice.2025.10310

**Published:** 2025-12

**Authors:** Yuanli Lei, Maria Alkozah, Rita Wilson Dib, Bibi Maryam, Brandon Mohler, Emily A. Siegrist, Joseph Sassine

**Affiliations:** 1 Hospital Medicine Section, Department of Internal Medicine, University of Oklahoma Health Sciences Center, Oklahoma City, OK, USA; 2 Infectious Diseases Section, Department of Internal Medicine, University of Oklahoma Health Sciences Center, Oklahoma City, OK, USA; 3 Internal Medicine Residency Program, Department of Internal Medicine, University of Oklahoma Health Sciences Center, Oklahoma City, OK, USA; 4 Oklahoma Clinical and Translational Science Institute, University of Oklahoma Health Sciences Center, Oklahoma City, OK, USA; 5 Department of Pharmacy, University of Oklahoma Health Sciences Center, Oklahoma City, OK, USA

## Abstract

This single-center retrospective analysis evaluated the yield of blood cultures in patients with febrile neutropenia during a supply shortage. The detection rate of true bacteremia was observed to increase with the number of sets obtained, although this increase was not statistically significant. Findings support limiting repeat cultures within 48 hours.

## Introduction

Bacteremia occurs in up to 23–29% of febrile neutropenia (FN) episodes.^1,2^ The National Comprehensive Cancer Network, Infectious Diseases Society of America, and American Society for Microbiology recommend obtaining at least two and up to four sets of blood cultures from different anatomic sites at the onset of FN.^3,4^ The collection of two sets, instead of one, is associated with an increased sensitivity for detecting bloodstream infections.^5^ It also allows better discrimination between true pathogens and contaminants, thereby improving the diagnostic yield, which is critical in guiding appropriate antimicrobial therapy in immunocompromised patients.^6^

A recent manufacturing interruption of blood culture bottles compatible with the BACTEC system, which began in June 2024, led to the implementation of conservative measures, including an institutional restriction on the number and frequency of blood culture collections. We herein sought to determine the impact of this shortage on the blood culture yield for FN, using real-world data.

## Methods

This is a single-center, retrospective, observational study evaluating adult patients with neutropenic fever who had blood cultures collected within the Oklahoma University Medical Center, from April 1, 2024, through November 30, 2024. An episode of FN was defined as a single oral temperature greater than or equal to 101°F (38.3°C) or a temperature greater than or equal to 100.4°F (38°C) sustained over a 1-hour period and an absolute neutrophil count (ANC) < 0.5*10^3^/uL.^3^ Febrile episodes during the study period that were separated by at least 7 days were considered as distinct episodes. Blood cultures collected within 7 days after the index blood culture was obtained were considered follow-up blood cultures. Episodes occurring between April 1, 2024, and July 31, 2024, were labeled to have occurred during the “Liberal period.” During that period, there were no restrictions on blood culture orders by number or frequency. Episodes occurring between August 1, 2024, and November 30, 2024, were labeled to have occurred during the “Restriction period.” In response to the blood culture shortage that occurred during that period, the hospital system implemented changes to conserve the limited supply, which came into effect on August 1, 2024. Hard stops on blood culture ordering in the electronic medical record were placed to limit ordering to one blood culture set, including an aerobic and anaerobic bottle, every 48 hours per patient. No restrictions were placed on the volume of collected blood per bottle. Exceptions were allowed per clinical scenario as assessed and approved by on-call Infectious Diseases specialists. Basic demographic and clinical data were collected. The primary outcome evaluated was the prevalence of bacteremia in patients with FN between the Liberal period and the Restriction period. The secondary outcome was the diagnostic yield of blood cultures depending on the number of blood culture sets obtained, using a previously described definition.^5^ Episodes resulting in a single positive set blood culture for commensal microbes (coagulase-negative *Staphylococcus*, *Gemella* spp., *Corynebacterium* spp., *Rothia* spp.) were considered contaminants. Chi-square and Mann–Whitney U tests were used to compare groups as appropriate. Statistical analysis was performed on IBM SPSS version 29.0.0.0.

## Results

A total of 236 episodes of FN were identified during the study period (134 episodes during the Liberal period, and 102 episodes during the Restricted period), involving a total of 173 patients (Table S1). The median temperature, ANC, and presence of central venous catheter were comparable between the two groups. There was no significant difference in the rate of detected bacteremia on the initial blood culture between the Liberal and Restricted periods (30.6% vs 24.5%, *P* = 0.3). Follow-up blood cultures were more often obtained during the Liberal period (57.5% vs 52.0%) with similar rates of positivity (15.6% vs 9.4%, *P* = 0.2). Only 6.5% (Liberal period) and 7.5% (Restriction period) of follow-up blood cultures detected a different organism than the initial culture (Table 1). Higher ANC was associated with significantly lower rates of microbial growth on univariate and multivariate analysis (odds ratio 0.004 for every 1k/µL increase in ANC, 95% confidence interval: 0–0.09, *P* < 0.001). No difference in microbial growth was noted when comparing blood cultures obtained during Restriction and Liberal periods (Table S2).

**Table 1.** Characteristics of Neutropenic Fever Episodes and Blood Culture Results

Characteristic	Liberal period (n = 134)	Restriction period (n = 102)	Total episodes (n = 236)	***P*** **-value**
Documented temperature in Celsius, median (IQR)	38.3 (38–38.8)	38.3 (37.9–38.9)	38.3 (38–38.9)	0.982
Absolute neutrophil count in k/µL, median (IQR)	0.02 (0–0.14)	0.02 (0–0.19)	0.02 (0–0.16)	0.881
Central venous catheter present	101 (75.4%)	69 (67.6%)	170 (72%)	0.190
*Number of initial blood cultures collected*				< 0.001
1	11 (8.2%)	96 (94.1%)	107 (45.3%)	
2	83 (61.9%)	6 (5.9%)	89 (37.7%)	
3	23 (17.2%)	0 (0%)	23 (9.7%)	
4	15 (11.2%)	0 (0%)	15 (6.4%)	
5	1 (0.7%)	0 (0%)	1 (0.4%)	
6	1 (0.7%)	0 (0%)	1 (0.4%)	
Microbial growth on initial blood cultures	41 (30.6%)	25 (24.5%)	66 (28%)	0.302
*Number of positive initial blood culture bottles*				< 0.001
1	13/41 (31.7%)	25/25 (100%)	38/66 (57.6%)	
2	24/41 (58.5%)	–	24/66 (36.4%)	
3	2/41 (4.9%)	–	2/66 (3%)	
4	2/41 (4.9%)	–	2/66 (3%)	
*Isolated organism*				0.143
Anaerobes	5/41 (12.2%)	0/25 (0%)	5/25 (7.6%)	
*Candida* species	0/41 (0%)	2/25 (8%)	2/25 (3%)	
Coagulase-negative *Staphylococcus*	6/41 (14.6%)	2/25 (8%)	8/25 (12.1%)	
*Escherichia coli*	7/41 (17.1%)	2/25 (8%)	9/25 (13.6%)	
*Enterococcus* species	5/41 (12.2%)	7/25 (28%)	12/25 (18.2%)	
Non-fermenter Gram-negative	2/41 (4.9%)	0/25 (0%)	2/25 (3%)	
Other *Enterobacterales*	3/41 (7.3%)	1/25 (4%)	4/25 (6.1%)	
Other Gram-positive	5/41 (12.2%)	2/25 (8%)	7/25 (10.6%)	
*Pseudomonas aeruginosa*	1/41 (2.4%)	3/25 (12%)	4/25 (6.1%)	
*Staphylococcus aureus*	2/41 (4.9%)	1/25 (4%)	3/25 (4.5%)	
*Streptococcus* species	5/41 (12.2%)	5/25 (20%)	10/25 (15.2%)	
*Number of follow-up blood cultures in the same episode*				< 0.001
0	57 (42.5%)	49 (48%)	106 (44.9%)	
1	10 (7.5%)	42 (41.2%)	52 (22%)	
2	25 (18.7%)	5 (4.9%)	30 (12.7%)	
3	9 (6.7%)	4 (3.9%)	13 (5.5%)	
4	13 (9.7%)	2 (2%)	15 (6.4%)	
5 or more	20 (14.9%)	0 (0%)	20 (8.5%)	
*Result of follow-up blood culture*				0.242
No growth	65/77 (84.4%)	48/53 (90.6%)	113/130 (86.9%)	
Growth of the same organism	7/77 (9.1%)	1/53 (1.9%)	8/130 (6.2%)	
Growth of a different organism	5/77 (6.5%)	4/53 (7.5%)	9/130 (6.9%)	

When stratified by number of initial blood culture sets obtained, the diagnostic yield of blood culture for bacteremia increased with the number of blood culture sets obtained without reaching statistical significance (19.6%, 25.8%, 30.4%, 46.6% for 1, 2, 3, 4 sets of blood cultures, respectively, *P* = 0.12), including when the yield of one set was compared to the yield of two sets (*P* = 0.29) (Table 2).

**Table 2.** Diagnostic Yields of Initial Blood Cultures Stratified by Number of Sets

Number of blood culture sets collected	Total episodes	Episodes with true bacteremia	Diagnostic yield (%)	*P*-value
**1**	107	21	19.6	0.12
**2**	89	23	25.8	
**3**	23	7	30.4	
**4**	15	7	46.6	
**5**	1	0	0.0	
**6**	1	0	0.0	

## Discussion

Our study found no significant difference in the rate of microbial growth on initial blood cultures for patients with FN between the Liberal and Restricted periods. We also noted a trend of increasing diagnostic yield of blood cultures with more sets of blood at the onset of a febrile neutropenic episode, although not statistically significant. Anaerobes, coagulase-negative *Staphylococcus*, and *Escherichia coli* appear to have a lower growth rate on initial cultures during the Restriction period.

Interestingly, these results are not concordant with the literature evaluating cohorts of non-neutropenic patients, where higher positivity rates were observed with paired-set blood cultures compared with a single set.^7^ Their study also noted a higher rate of Gram-negative growth and a lower rate of Gram-positive growth on cultures, when compared with our study. When studying the need for peripheral sticks in 241 pediatric patients with FN and bacteremia, the authors found that 15% of the positive blood cultures originated from the central line, while 24% came from the peripheral site alone, suggesting a risk of missed diagnoses if only a single culture were obtained.^8^

Repeat blood culture yield was low in both of our cohorts. Despite that, fewer repeat blood culture sets with longer intervals were used in the Restricted period. Serody et al. showed that among hematopoietic transplant recipients with FN, the prevalence of positive blood culture after day one of fever was low at 4.6%, and most repeat cultures demonstrated the same organism as the initial cultures.^9^ Such findings have been reproduced by other studies.^10^

Based on our study findings and evidence from the literature, our institute is continuing the policy limiting repeat blood cultures with intervals less than 48 hours, while resuming two sets of blood cultures after the shortage ended.

Our analysis has some limitations. Being retrospective allows for potential biases. The relatively small number of episodes may have contributed to the limited power of our study to find statistical significance among the compared groups. Additionally, we did not have data on total blood volume collected, which is known to affect the diagnostic yield of blood cultures.

In conclusion, this blood culture bottle shortage inadvertently created opportunities for diagnostic stewardship in patients with FN, particularly in limiting the use of low-yield follow-up blood cultures. Future studies with a larger sample size and controlled designs with a focus on neutropenic patients are needed to determine the optimal number of blood culture bottles in FN.

## Supporting information

Lei et al. supplementary material Lei et al. supplementary material

## Supplementary material

To view supplementary material for this article, please visit https://doi.org/10.1017/ice.2025.10310.
