# Supplementary material for: Diagnostic yield of blood cultures in febrile neutropenia—a real-world observational study from an academic medical center during blood culture bottle shortage
Source: Infect Control Hosp Epidemiol. 2025 Oct 10;46(12):1278–81. doi: 10.1017/ice.2025.10310 (PMC12779455; doi:10.1017/ice.2025.10310)
Supplement: Lei et al. supplementary material [file S0899823X25103103sup001.docx]

Supplement

**Table S1 – Patient Characteristics**

| **Characteristic** | **Total Population (n=173)** |
| --- | --- |
| Age in years, median (IQR) | 55 (40-66) |
| *Sex* |  |
| Female | 87 (50.3%) |
| Male | 86 (49.7%) |
| *Race/Ethnicity* |  |
| African American | 23 (13.3%) |
| Asian | 3 (1.7%) |
| Hispanic | 10 (5.8%) |
| Native American | 12 (6.9%) |
| White | 125 (72.3%) |
| *Underlying disease* |  |
| Acute lymphoblastic leukemia (ALL) | 15 (8.7%) |
| Acute myeloid leukemia (AML) | 60 (34.7%) |
| Lymphoma | 30 (17.3%) |
| Myelodysplastic syndrome (MDS) | 7 (4%) |
| Multiple myeloma | 15 (8.7%) |
| Solid tumor | 30 (17.3%) |
| Other | 16 (9.2%) |
| Prior autologous HCT | 15 (8.7%) |
| Prior allogeneic HCT | 14 (8.1%) |
| Prior CAR T cell therapy | 14 (8.1%) |

**Table S2 – Predictors of Microbial Growth on Initial Blood Culture by Logistic Regression**

| **Predictor** | **Univariate** | | | **Multivariate** | | |
| --- | --- | --- | --- | --- | --- | --- |
|  | **OR** | **95% CI** | **p-value** | **aOR** | **95% CI** | **p-value** |
| Documented temperature in Celsius | 1.33 | 0.94-1.86 | 0.105 | 1.26 | 0.87-1.83 | 0.218 |
| Absolute neutrophil count in k/µL | 0.003 | 0-0.07 | < 0.001 | 0.004 | 0-0.09 | < 0.001 |
| *Blood culture period* |  |  |  |  |  |  |
| Liberal period | Ref. |  |  | Ref. |  |  |
| Restriction period | 0.74 | 0.41-1.32 | 0.303 | 0.74 | 0.40-1.35 | 0.319 |
